# The evaluation of the reduction of radiation dose via deep learning-based reconstruction for cadaveric human lung CT images

**DOI:** 10.1038/s41598-022-16798-9

**Published:** 2022-07-20

**Authors:** Tomo Miyata, Masahiro Yanagawa, Noriko Kikuchi, Kazuki Yamagata, Yukihisa Sato, Yuriko Yoshida, Mitsuko Tsubamoto, Noriyuki Tomiyama

**Affiliations:** 1 Department of Future Diagnastic Radiology, Osaka University Graduate School of Medicine, 2-2 Yamadaoka Suita-City, Osaka, 565-0871 Japan; 2 Department of Radiology, Osaka University Graduate School of Medicine, 2-2 Yamadaoka, Suita-city, Osaka, 565-0871 Japan; 3 Department of Radiology, Suita Municipal Hospital, 5-7 Kishibeshinmati, Suita-city, Osaka, 564-8567 Japan; 4 Department of Radiology, Nishinomiya Municipal Central Hospital, 8-24 Hayashidacho, Nishinomiya City, Hyogo 663-8014 Japan

**Keywords:** Machine learning, Imaging, Image processing

## Abstract

To compare the quality of CT images of the lung reconstructed using deep learning-based reconstruction (True Fidelity Image: TFI ™; GE Healthcare) to filtered back projection (FBP), and to determine the minimum tube current–time product in TFI without compromising image quality. Four cadaveric human lungs were scanned on CT at 120 kVp and different tube current–time products (10, 25, 50, 75, 100, and 175 mAs) and reconstructed with TFI and FBP. Two image evaluations were performed by three independent radiologists. In the first experiment, using the same tube current–time product, a side-by-side TFI and FBP comparison was performed. Images were evaluated with regard to noise, streak artifacts, and overall image quality. Overall image quality was evaluated in view of whole image quality. In the second experiment, CT images reconstructed using TFI and FBP with five different tube current–time products were displayed in random order, which were evaluated with reference to the 175 mAs-FBP image. Images were scored with regard to normal structure, abnormal findings, noise, streak artifacts, and overall image quality. Median scores from three radiologists were statistically analyzed. Quantitative evaluation of noise was performed by setting regions of interest (ROIs) in air. In first experiment, overall image quality was improved, and noise was decreased in images of TFI compared to that of FBP for all tube current–time products. In second experiment, scores of all evaluation items except for small vessels in images of 25 mAs-TFI were almost the same as that of 175 mAs-FBP (all *p* > 0.31). Using TFI instead of FBP, at least 85% radiation dose reduction could be possible without any degradation in the image quality.

## Introduction

Recently, deep learning has been applied in many fields of life sciences and medicine, such as cellular image analysis or pharmacogenomics^1,2^. Additionally, it has also been used in image reconstruction^3^ and it is reported that image reconstruction using deep learning improves the image quality of computed tomography (CT), such as coronary artery or abdominal ultra-high-resolution CT (U-HRCT) images^3,4^. Several investigations have revealed the risk of CT-related radiation exposure, especially in younger patients, over the past decades^5–8^. However, reducing the CT radiation dose increases noise, degrades image quality, and in turn affects diagnosis^9^. CT manufacturers have developed image reconstruction methods that reduce the noise in noisy CT images acquired from CT data scanned at lower radiation doses. As a result, the radiation dose can be reduced^10^. However, it has been reported that excessive use of conventional IR algorithms may cause blotchy images, which are inappropriate for diagnosis compared to FBP images^11^. Therefore, a reconstruction method that reduces noise while maintaining image quality similar to that of an FBP image is required.

Recently, there have been several reports of image reconstruction methods using deep learning that enable noise reduction. For example, Canon announced the deep leaning-based reconstruction (DLR) method AiCE (Advanced Intelligent Clear-IQ Engine). It has been reported that this DLR provides better image quality and lesion detection than conventional iterative reconstruction and filtered back projection (FBP) images at low-dose chest and abdominopelvic CT^12^. The same report determined that DLR reduces noise, as a result, it is possible to reduce radiation doses without any degradation in the overall image quality. True Fidelity Image (TFI), a DLR employing filtered back projection (FBP) as training data, was announced by General Electric (GE) Healthcare. This image reconstruction specially reduces noise without degrading image quality^13^. Therefore, by using this reconstruction method we can expect to obtain clearer CT images with less radiation dose than before.

However, there is no report on how much the radiation dose can be reduced using TFI without any degradations in the overall image quality and an increase in the noise. Therefore, we hypothesized that using TFI could reduce the radiation dose ever before without degrading the image quality. The purpose of this study was to compare the quality of CT images of the lung reconstructed using TFI to FBP, and to determine the minimum tube current–time product in TFI without compromising the image quality.

## Materials and methods

This study was approved by the internal Ethics Review Board of Osaka University (Ethical approval number: 18417). Informed consent for the retrospective review of patient records and images and use of patient biomaterial was waived. All study procedures were conducted in accordance with the guidelines and regulations of our internal Ethics Review Board.

## Cadaveric human lungs

Four cadaveric lungs used in this study were pathologically proven as diffuse alveolar hemorrhage (*n* = 2), diffuse pan bronchiolitis (*n* = 1), and usual interstitial pneumonia (*n* = 1). The lungs were inflated and fixed using Heintzman’s method^14^, i.e., they were distended through the main bronchus with a fixative fluid containing polyethylene glycol 400, 95% ethyl alcohol, 40% formalin, and water with a 10:5:2:3 proportion. The specimens were immersed in the fixative fluid for 2 days and then air-dried.

## Image acquisition

The cadaveric lungs were fixed inside the chest phantom (Lung Man; Kyoto Kayaku) and scanned using a CT (Revolution CT ™; General Electronics) at 120 kVp and different tube current–time products (10, 25, 50, 75, 100, and 175 mAs). FOV was 200 mm and matrix size was 512 × 512. 175 mAs was maximum tube current–time products in our institute. The CT dose indices were 0.68, 1.7, 3.4, 5.2, 6.8, and 11.9 mGy. Each cadaveric lung was scanned in one slice at three different position levels. So we acquired three images per cadaveric lung. Scan mode was helical scan and acquired CT images were axial image.

**Figure 1:**
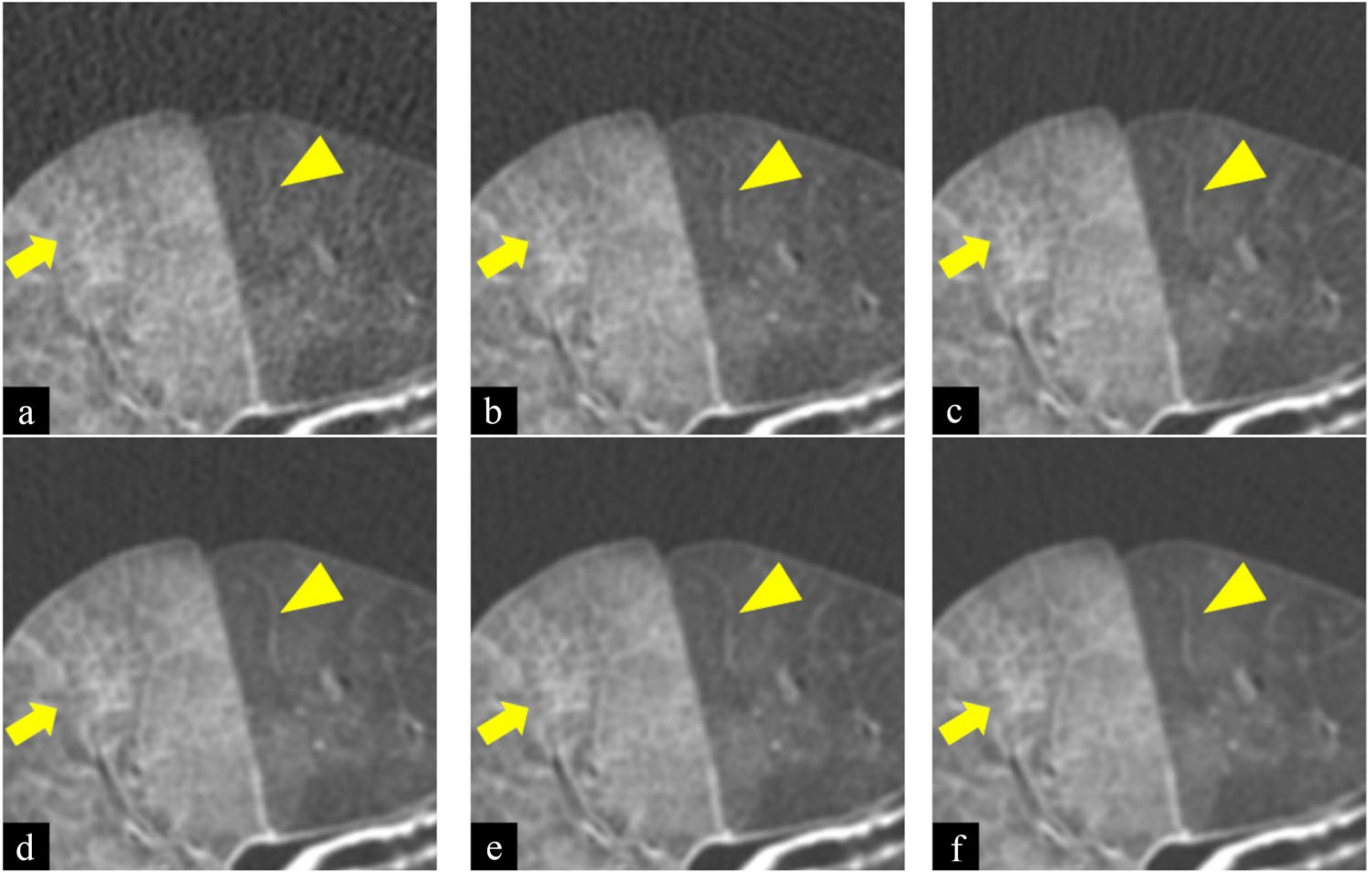
Images of filtered back projection (FBP) of cadaveric lung (diffuse alveolar hemorrhage) at 10 (**a**), 25 (**b**), 50 (**c**), 75 (**d**), 100 (**e**), and 175mAs (**f**). Window width was 1200 and Window level was − 700 (Hounsfield Unit). Scan field of view was 20 cm. The length and width of each figure was 6.5 cm. As tube current–time product decreased, the shape of the small vessel (arrowhead) and reticulation (arrow) became obscured. In addition, noise and streak artifacts increased.

**Figure 2:**
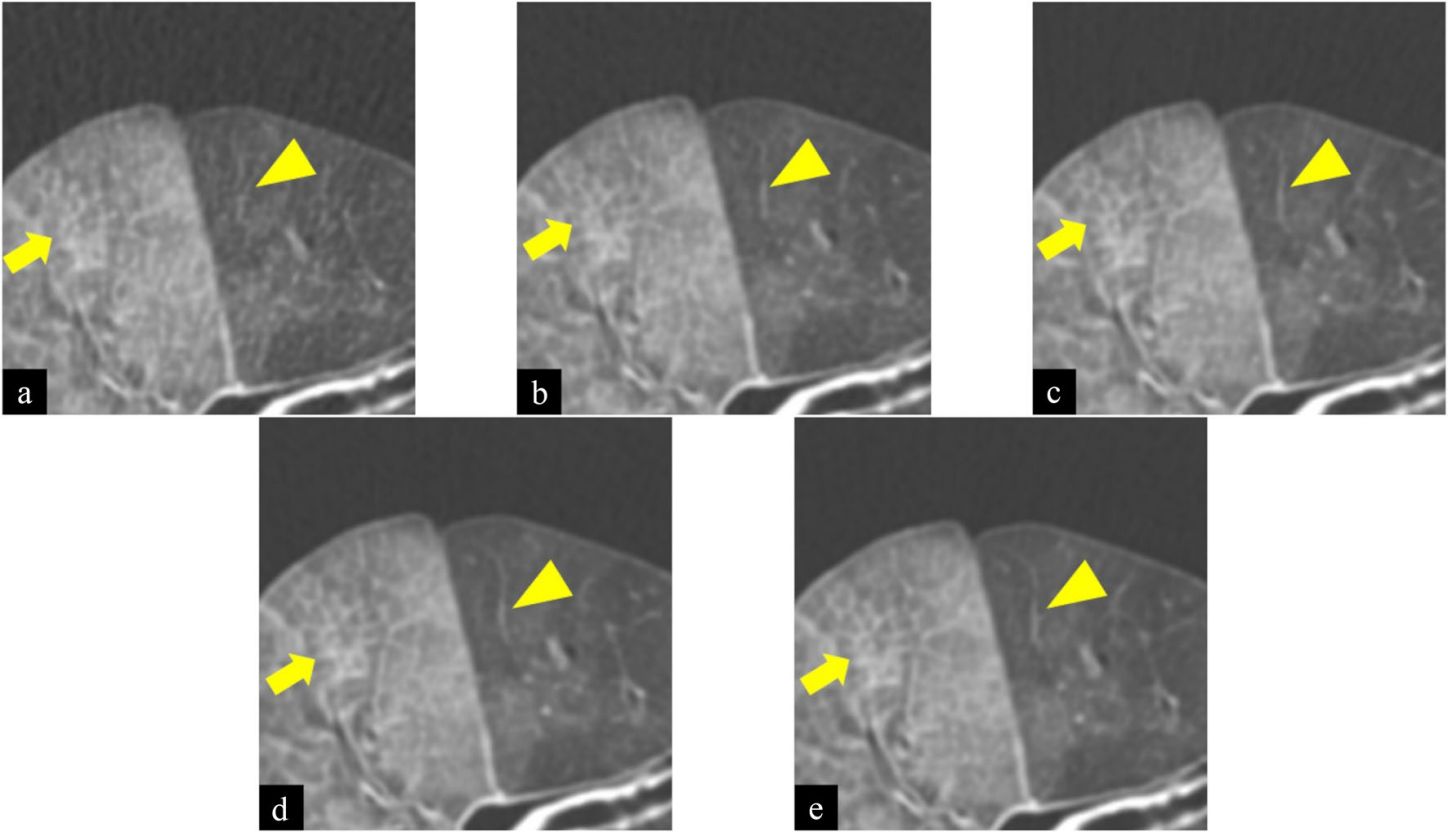
Images of True Fidelity Image (TFI) of cadaveric lung (diffuse alveolar hemorrhage) at 10 (**a**), 25 (**b**), 50 (**c**), 75 (**d**), and 100 mAs (**e**). Window width was 1200 and Window level was − 700 (Hounsfield Unit). Scan field of view was 20 cm. The length and width of each figure was 6.5 cm. As tube current–time product decreased, the shape of the small vessel (arrowhead) and reticulation (arrow) became obscured. In addition, noise and streak artifacts increased.

All CT images with 0.625 mm slice thickness were reconstructed using FBP and TFI (medium setting) with standard kernel (Figs. 1 and 2). We obtained 24 images (12 images of FBP and 12 images of TFI).

## Determination of radiation dose reduction limit by evaluation of image quality

To determine the minimum tube current–time product in TFI without compromising the image quality, two types of image quality evaluation experiments were performed. In the first experiment, using the same tube current–time product, side-to-side comparison of TFI to FBP was performed by three independent radiologists who evaluated the noise, streak artifact, and overall image quality. In the second experiment, CT images reconstructed using FBP and TFI with five different tube current–time products were displayed in random order, which were evaluated by three independent radiologists compared to the 175 mAs-FBP image. Scoring for noise and streak artifact are described in Table 1. Scoring for overall image quality are described in Table 2. The monitor used in the image quality evaluation experiments was an 8.3megapixel 32inch color liquid crystal display monitor.

**Table 1:** Scoring for noise and streak artifact.

Score 1	Very low
Score 2	Low
Score 3	Fair
Score 4	Severe
Score 5	Extremely severe

**Table 2:** Scoring for overall image quality.

Score 1	Very poor (it is very difficult to detect structures and clearly evaluate their margins or internal properties)
Score 2	Poor (detection of structures is possible but it is difficult to clearly evaluate their margins or internal properties)
Score 3	Fair (margins or internals properties can be detected and are not inferior to the reference image)
Score 4	Better (detection of structures and the evaluation of their margins or internal properties was easier compared to the reference image)
Score 5	Excellent (detection of structures and the evaluation of their margins or internal properties was significantly easier compared to the reference image and there was no indistinct findings)

## Comparison of TFI and FBP image quality with the same tube current–time product

In the first evaluation, we compared the overall image quality, noise, and streak artifact of TFI and FBP. We selected an image obtained with the TFI reconstruction and the corresponding FBP image, at the same tube current–time product and slice level, and evaluated noise (*n* = 12), streak artifact (*n* = 12), and overall image quality (*n* = 12). Tube current–time product of images used for the evaluation were 10, 25, 50, 75, and 100 mAs. We displayed two images side-by-side. The side and image presentation order were randomly assigned. The image displayed on the right side was evaluated with reference to the image displayed on the left side. The overall image quality was evaluated with regard to the whole image. Three radiologists (with 4, 9, and 18 years of experience) independently scored the images with respect to the reference images using a 5point scale.

## Comparison of 175 mAs-FBP images with TFI and FBP images of the other tube current–time product

In the second evaluation, we evaluated the image quality of TFI and FBP with five different tube current–time products (10, 25, 50, 75, and 100 mAs) compared to images of 175 mAs-FBP. We chose an image randomly from the five aforementioned tube current–time products of TFI or FBP. Three independent radiologists evaluated the selected image with reference to the image with 175 mAs-FBP in the same case and at the same level. Evaluation was performed on the following items: small vessel (*n* = 82), bronchi (*n* = 47), bronchiectasis (*n* = 84), ground-glass opacity (*n* = 36), reticulation (*n* = 14) and solid nodule (*n* = 32). To obtain uniform evaluation points, these items were marked in advance. In addition, overall image quality (*n* = 12), noise (*n* = 12), and streak artifact (*n* = 12) were evaluated. These items were evaluated on the complete image. Three radiologists scored on the same 5point scale used in the first evaluation. The scores of all evaluation items for the 175 mAs-FBP used for reference were set to 3 point.

## Quantitative evaluation of noise

We evaluated image noise quantitatively. We set circular regions of interest (ROIs) on the air portion of images and measured the standard deviation (SD) values of ROIs using the workstation. We used the same monitor as in the aforementioned evaluations. Noise can be quantitatively evaluated by measuring the standard deviation (SD) value with an ROI (φ20mm) in the air portion. We set three ROIs of the same size at the same location on the selected FBP and TFI images with five different tube current–time products (10, 25, 50, 75, and 100 mAs) and FBP images with 175 mAs. Then, the average SD values of three ROIs were calculated.

## Statistical analysis

In the first evaluation, the median scores given by three radiologists and the statistical significance of the differences among five different tube current–time products were analyzed using the Shapiro–Wilk test to test normality and Mann–Whitney’s *U* test. *p* < 0.05 was considered significant. In the second evaluation, the median scores given by three radiologists and the statistical significance of the differences among five different tube current–time products were analyzed using the Shapiro–Wilk test to test normality and two-way ANOVA with Bonferroni post hoc test. *p* < 0.005 was considered significant. In the quantitative noise evaluation, two-way ANOVA and Bonferroni post hoc test were used to compare the noise of FBP and TFI images at five different tube current–time products (10, 25, 50, 75, and 100 mAs) with the noise of 175 mAs-FBP images. The results of the normality test were nonparametric in all evaluations. SPSS (version 24; IBM) was used for statistical analysis. *p* < 0.005 was considered significant.

## Results

### Comparison of TFI and FBP image quality at the same tube current–time product

Scores and the results of the comparisons are summarized in Table 3. TFI showed better scores than FBP for all tube current–time products in terms of overall image quality (all *p* < 0.001). In terms of noise, TFI presented lower noise than FBP for all tube current–time products (all *p* < 0.001). As for streak artifact, the scores of the TFI were better than those of the FBP at 25, 50, 75, and 100 mAs (all *p* < 0.001), but they were almost the same at 10 mAs.

**Table 3:** Subjective comparison of TFI and FBP at the same tube current–time products: CT findings.

Tube current time products (mAs)	Score					
		10	25	50	75	100
Noise	TFI	1.2 ± 0.4*	1.7 ± 0.5*	2.0 ± 0.0*	2.3 ± 0.4*	2.0 ± 0.0*
	FBP	4.8 ± 0.4*	4.5 ± 0.5*	3.8 ± 0.4*	3.5 ± 0.5*	3.6 ± 0.5*
Streak artifact	TFI	3.0 ± 0.0	2.3 ± 0.5*	2.1 ± 0.3*	1.8 ± 0.4*	2.1 ± 0.3*
	FBP	3.0 ± 0.0	3.5 ± 0.5*	4.3 ± 0.4*	3.7 ± 0.5*	3.8 ± 0.4*
Overall image quality	TFI	4.2 ± 0.4*	4.0 ± 0.0*	4.0 ± 0.0*	4.0 ± 0.0*	4.0 ± 0.0*
	FBP	1.8 ± 0.4*	1.8 ± 0.4*	2.0 ± 0.0*	2.5 ± 0.5*	2.4 ± 0.5*

Scores are presented as mean ± SD. Scores of subjective image analysis were statistically analyzed using the Mann–Whitney’s *U* test.

*There was a significant difference between TFI and FBP at the same tube current–time products (*p* < 0.05).

## Comparison of 175-mAs-FBP images with TFI and FBP images of the other tube current–time product

**Figure 3:**
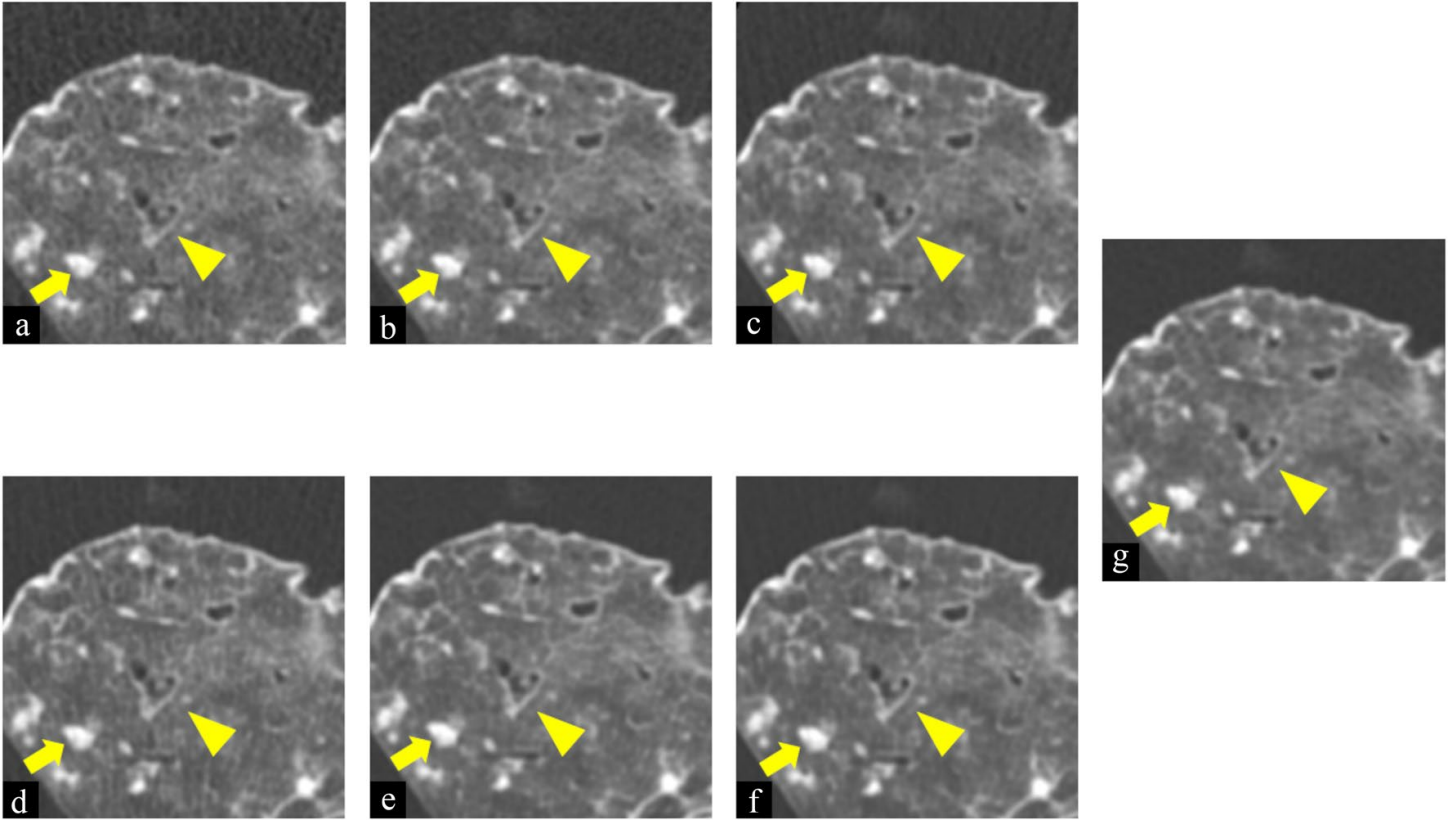
Images of filtered back projection (FBP) at 10 (**a**), 25 (**b**), 50 (**c**), and 175 mAs (**g**) and True Fidelity Image (TFI) at 10 (**d**), 25 (**e**), and 50 mAs (**f**) of cadaveric lung (usual intestinal pneumonia). Window width was 1200 and Window level was − 700 (Hounsfield Unit). Scan field of view was 20 cm. The length and width of each figure was 6.5 cm. As tube current–time product decreased, the shape of the nodule (arrowhead) and bronchiectasis (arrow) at TFI and FBP varied slightly or were almost the same as that of 175 mAs-FBP.

The scores and comparison results of TFI are summarized in Table 4 and those of FBP are summarized in Table 5 (two-way ANOVA analyzing the interaction of reconstruction methods and tube current–time products: F > 4.9, *p* < 0.001). In evaluations of items such as normal structures (small vessel and bronchi) and abnormal findings (ground-glass opacity, bronchiectasis, reticulation, and nodule), scores of 10mAs-TFI were worse than that of 175 mAs-FBP for small vessels, bronchi, and reticulation (all *p* < 0.001). The scores of 10 mAs-TFI were not significantly different compared to that of 175 mAs-FBP for ground-glass opacity, bronchiectasis, and nodule (Fig. 3). Scores of 25, 50, 75, and 100 mAs-TFI were significantly better (all *p* < 0.001) or not significantly different compared to that of 175 mAs-FBP for normal structures and abnormal findings. As for noise, compared to 175 mAs-FBP, noise was significantly increased at 10mAs of TFI. At 25 and 50 mAs-TFI (all *p* < 0.001), noise was almost the same as that of 175 mAs-FBP. Noise was significantly decreased at 75 and 100 mAs-TFI (all *p* < 0.001). As for the streak artifact, compared to that of 175 mAs-FBP, streak artifact was significantly increased at 10 mAs-TFI (*p* < 0.001). At the other tube current–time products, streak artifact was not significantly different from that of 175 mAs-FBP. As for overall image quality, scores of 10 and 25 mAs-TFI were almost the same as that of 175 mAs-FBP. Scores of 50, 75, and 100 mAs-TFI were significantly better than that of 175 mAs-FBP (all *p* < 0.001). In particular, 25 mAs-TFI was comparable to 175 mAs-FBP without any degradation in the image quality for all items.

**Table 4:** Subjective evaluation of TFI compared to 175-mAs-FBP: CT findings.

Tube current–time products (mAs)	Score				
	10	25	50	75	100
Bronchi	2.3 ± 0.1*	2.9 ± 0.1	3.0 ± 0.1	3.0 ± 0.1	3.3 ± 0.1*
Small vessel	2.6 ± 0.1*	3.7 ± 0.1*	3.8 ± 0.1*	4.0 ± 0.1*	4.3 ± 0.1*
Bronchiectasis	2.9 ± 0.0	3.0 ± 0.0	3.2 ± 0.0*	3.1 ± 0.0	3.4 ± 0.0*
Ground-glass Opacity	2.8 ± 0.1	3.2 ± 0.1	3.1 ± 0.1	3.0 ± 0.1	3.2 ± 0.1
Reticulation	2.5 ± 0.1*	3.2 ± 0.1	3.7 ± 0.1*	3.6 ± 0.1*	3.7 ± 0.1*
Nodule	3.1 ± 0.1	3.0 ± 0.1	3.0 ± 0.1	3.0 ± 0.1	3.1 ± 0.1
Noise	4.0 ± 0.1*	3.0 ± 0.1	2.8 ± 0.1	2.2 ± 0.1*	2.0 ± 0.1*
Streak artifact	4.0 ± 0.1*	3.5 ± 0.1	2.8 ± 0.1	2.3 ± 0.1	2.3 ± 0.1
Overall image Quality	2.5 ± 0.1	3.4 ± 0.2	3.8 ± 0.1*	3.8 ± 0.1*	4.0 ± 0.1*

Scores are presented as mean ± SD. Scores were statistically analyzed using two-way ANOVA and the Bonferroni test as post-hoc test to compare the scores of TFI images at five different tube current–time products (10-, 25-, 50-, 75-, and 100-mAs) with the scores of 175-mAs-FBP images.

Scores of 175-mAs-FBP are 3 point.

*There was a significant difference between TFI and 175-mAs-FBP (*p* < 0.005).

**Table 5:** Subjective evaluation of FBP compared to 175-mAs-FBP: CT findings.

Tube current–time products (mAs)	Score				
	10	25	50	75	100
Bronchi	1.9 ± 0.1*	2.7 ± 0.1	2.9 ± 0.1	2.9 ± 0.1	3.0 ± 0.1
Small vessel	1.8 ± 0.1*	2.5 ± 0.1*	2.8 ± 0.1	3.0 ± 0.1	3.3 ± 0.1
Bronchiectasis	2.6 ± 0.0*	2.9 ± 0.0	3.0 ± 0.0	3.0 ± 0.0	3.0 ± 0.0
Ground-glass Opacity	2.5 ± 0.1*	2.8 ± 0.1	2.9 ± 0.1	3.1 ± 0.1	3.1 ± 0.1
Reticulation	1.8 ± 0.1*	2.7 ± 0.1	3.0 ± 0.1	3.1 ± 0.1	3.1 ± 0.1
Nodule	2.7 ± 0.1	3.0 ± 0.1	3.0 ± 0.1	3.2 ± 0.1	3.0 ± 0.1
Noise	5.0 ± 0.1*	4.6 ± 0.1*	4.0 ± 0.1*	3.6 ± 0.1	3.5 ± 0.1
Streak artifact	3.7 ± 0.1	3.8 ± 0.1*	4.0 ± 0.1*	3.4 ± 0.1	3.5 ± 0.1
Overall image Quality	1.1 ± 0.1*	1.8 ± 0.2*	2.4 ± 0.1	2.8 ± 0.1	2.8 ± 0.1

Scores are presented as mean ± SD. Scores were statistically analyzed using two-way ANOVA and the Bonferroni test as post-hoc test to compare the scores of FBP images at five different tube current–time products (10-, 25-, 50-, 75-, and 100-mAs) with the scores of 175-mAs-FBP images.

Scores of 175-mAs-FBP are 3 points.

*There was a significant difference between FBP and 175-mAs-FBP (*p* < 0.005).

## Quantitative evaluation of noise

**Figure 4:**
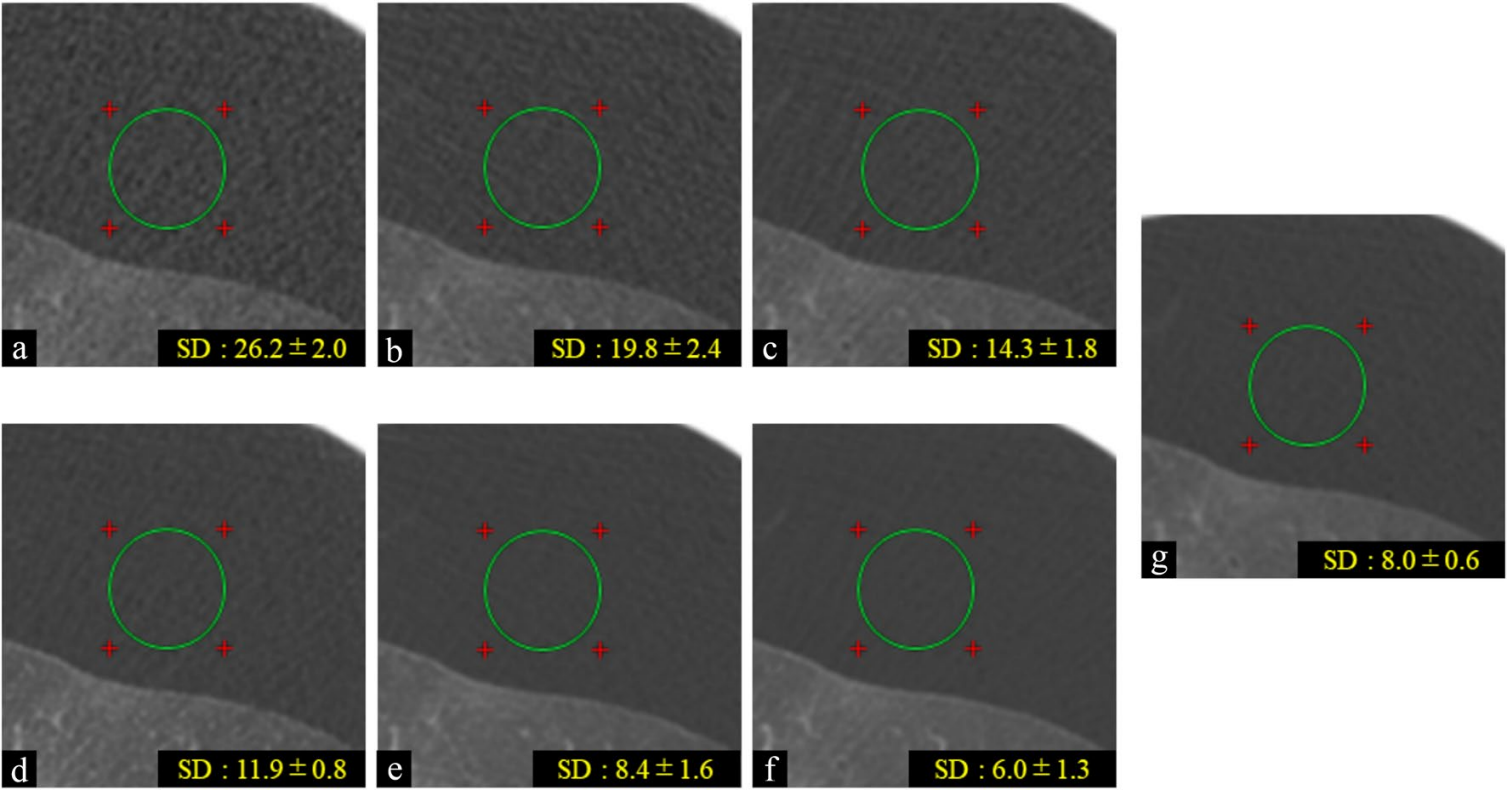
Images of filtered back projection (FBP) at 10 (**a**), 25 (**b**), 50 (**c**), and 175 mAs (**g**) and True Fidelity Image (TFI) at 10 (**d**), 25 (**e**), and 50 mAs (**f**) of cadaveric lung (diffuse alveolar hemorrhage). We set ROI of the same size at the same location on the selected FBP and TFI images in background air. The SD values of each image are presented. The SD values of 25 mAs-TFI were almost the same as that of 17 5 mAs-FBP.

The scores and comparison results are summarized in Table 6 (two-way ANOVA analyzing the interaction of reconstruction methods and tube current–time products: F > 226, *p* < 0.001). Noise of TFI and FBP was decreased as tube current–time products increased. At each tube current–time product, noise of TFI was decreased compared to that of FBP. This result corresponded to that of first subjective evaluation. Noise of 10-mAs-TFI was significantly increased compared to that of 175 mAs-FBP (*p* < 0.001). The noise of 25 mAs-TFI was almost the same as that of 175 mAs-FBP (Fig. 4). The noise of 50, 75, and 100 mAs-TFI was significantly decreased compared to that of 175 mAs-FBP (all *p* < 0.001).

**Table 6:** Quantitative evaluation of noise on TFI and FBP: SD value.

Tube current–time products (mAs)	Score					
	10	25	50	75	100	175 (FBP)
Noise (FBP)	26.2 ± 2.0*	19.8 ± 2.4*	14.3 ± 1.8*	11.9 ± 1.3*	10.6 ± 1.3*	8.0 ± 0.6
Noise (TFI)	11.9 ± 0.8*	8.4 ± 1.6	6.0 ± 1.3*	5.4 ± 0.9*	4.9 ± 0.7*	

SD values are presented as mean ± SD. SD values of quantitative evaluation of noise were statistically analyzed using two-way ANOVA and the Bonferroni test as post-hoc test to compare the noise of FBP and TFI images at five different tube current–time products (10-, 25-, 50-, 75-, and 100-mAs) with the noise of 175-mAs-FBP images.

*There was a significant difference compared to 175-mAs-FBP (*p* < 0.005).

## Discussion

The present study demonstrated that TFI had the potential to reduce noise and streak artifacts compared to FBP at the same tube current–time products and that TFI also had the potential not to deteriorate overall image quality regardless of dose reduction. TFI enabled at least 85% radiation dose reduction without any degradation in the image quality. TFI can reduce radiation exposure while preserving the image texture of FBP because it is a deep learning-based reconstruction technique on the basis of training data using FBP.

From the results of the first evaluation, it may be caused by reducing noise and streak artifact that TFI may provide the better image quality than FBP at the same tube current–time products. It has been reported that reduction of noise and streak artifacts might lead to an improvement in the overall image quality^15,16^. From the results of the second evaluation, scores of small vessel and reticulation at 50, 75, and 100 mAs-TFI were superior to those of 175 mAs-FBP. On the other hand, the scores of bronchi, ground-glass opacity, bronchiectasis, nodule, and streak artifact at 25, 50, 75, and 100 mAs-TFI were slightly or not significantly different from that of 175 mAs-FBP. We may speculate that the noise reduction mainly affects small structures, minute abnormal findings, and overall image quality. On the other hand, noise has little effect on large structures, bulky abnormal findings. Score of overall image quality at 50, 75, and 100 mAs-TFI were superior to those of 175 mAs-FBP. We may also speculate that clear visibility of small vessels and bronchial edges and reduction of noise and streak artifact lead to improvement of overall image quality. At 25 mAs-TFI, there was no difference between noise and streak artifacts, but the score of small vessels was better than that of 175 mAs-FBP. It is noted that deep learning reconstruction has the risk of creating imaginary objects^17^. In this study, no structures suspected of being created by TFI were observed. However, there may be possibility that the edges of small vessel were complemented by deep learning-based reconstruction. Hata et al. reported that small vessels might be obscured when using TFI with the standard kernel^13^. Thus, when using a deep learning-based reconstruction, care must be taken in evaluating small structures such as small vessels. It may be necessary to pathologically correlate with evaluations of small structures on CT images reconstructed by TFI in the future. Moreover, at 25 mAs-TFI, scores of items such as normal structures (small vessels and bronchi) and abnormal findings (ground-glass opacity, bronchiectasis, reticulation, and nodule) were almost the same or slightly better compared to 175 mAs-FBP. Noise, streak artifact, and overall image quality were almost the same in 25 mAs-TFI and 175 mAs-FBP. As for overall image quality, there was no significant difference between 10 mAs-TFI and 175 mAs-FBP. However, there were differences in some other evaluation items between 10 mAs-TFI and 175 mAs-FBP. The evaluation of quantitative noise at the 25 mAs-TFI was also in agreement with that at the 175 mAs-FBP. Therefore, 25 mAs-TFI is equal or slightly improved compared to 175 mAs-FBP without any increase in noise. Thus, TFI enabled to reduce at least 85% radiation dose without any degrading image quality. There are various reconstruction algorithms such as model-based iterative reconstruction (Veo: GE Healthcare, IMR: Philips Healthcare) or hybrid iterative reconstruction (ASIR; GE Healthcare, iDose4: Philips Healthcare, SAFIRE: Siemens Healthineers, AIDR3D: Canon Medical Systems)^10^. It has been reported that radiation dose can be reduced further with model-based IR compared to hybrid IR and FBP^10,18^. It was also reported that hybrid IR algorithms can reduce the radiation dose by 27–54% and model-based IR algorithms could reduce the radiation dose by up to 80%^19^. And it was reported that ASIR can reduce the radiation dose by nearly 80% compared to FBP without any degradations of overall image quality^20^. However, it was also reported that excessive use of conventional IR algorithms may cause blotchy image, resulting in obscuration of fine and subtle findings such as intralobular reticular opacities and peripheral vessels^11^. On the other hand, it was reported that deep learning-based reconstruction (DLR) algorithms have been developed and proved significant noise reductions without changing the typical noise texture of FBP images, therefore allowing dose reduction obtained by IR algorithms whilst preserving FBP image quality^21–23^. Therefore, the use of TFI do not result in blotchy images because TFI is a DLR constructed using FBP as training data. There have been several studies recommending screening with low-dose chest CT to reduce lung cancer mortality^24–27^. In addition, there is a report recommending low-dose chest CT in the detection and management of COVID-19^28^. These investigations indicate that, the demand for low-dose CT in screening is increasing. There are some studies showing the superiority of deep neural networks for low-dose CT^29,30^. Using TFI enables the reduction of radiation dose than ever before. While scanning with CT, we use the CT protocol according to the ALARA principle (as low as reasonably achievable)^31,32^. TFI may help optimize clinical protocols to meet the ALARA principle.

This study has some limitations. In spite of using a chest wall phantom, we cannot reproduce fluctuations of the chest wall. Furthermore, in this study, the effects of respiratory variation were not considered in the evaluation and it is debatable how applicable they are in clinical practice. Further study is needed to examine the influence of these fluctuations on the TFI. In this study, we applied images of 175 mAs-FBP as reference images to create a high quality image of the FBP. Naturally, this radiation dose is high, and might not be used for clinical diagnosis. The noise reduction rate is expected to depend on the dose used for scanning images. If a different reference dose level is chosen, the ratio of dose reduction allowed by TFI is likely to change. Only four cadaveric lung samples are also included in this study, thus limited anatomical variation and limited types of pathology. Radiologists may have been unknowingly influenced by other information, such as noise and streak artifacts, in their assessment of items such as normal structure and abnormal findings. Therefore, it is possible that the evaluation of these items may have depended on the degree of noise and streak artifacts. We evaluated TFI and FBP images using a side-by-side comparison. The radiologist’s evaluation may be influenced by this method. In this study, we used cadaveric lungs pathologically diagnosed. However, abnormal CT findings such as solid nodules were not correlated with pathological evaluation. We evaluated image noise quantitatively setting ROIs in background air. But noise in the background air may be not necessarily the same as noise in the tissue. Using noise reduction by deep learning algorithms can sharpen edges and make image clear^21–23^. However, we need to be careful because it may erase real structures or depict structures that do not exist^17^.

In conclusion, the TFI shows higher overall quality with lower noise and streak artifacts than the FBP at all tube current–time products. The result is a dose reduction of at least 85%. This may suggest that TFI has the potential to reduce radiation dose as much or more than conventional iterative reconstruction such as ASIR without degrading the overall image quality and losing the clarity of items such as normal structures and abnormal findings. This study only refers to experimental results using cadaveric lungs, and such results do not necessarily apply to actual clinical practice. Therefore, even in clinical practice, it is necessary to verify whether noise can be reduced as in this study and, as a result, verify if the radiation dose can be reduced and if imaginary objects are created. Thus, using TFI instead of FBP could help decrease the radiation dose while maintaining CT image quality, although further clinical studies are needed for actual clinical practice implementation.

## Supplementary Information

Supplementary Information 1.

Supplementary Information 2.

Supplementary Information 3.

Supplementary Information 4.

## Data Availability

All data generated or analyzed during this study are included in this published article and its supplementary information files.

## Abbreviations

CT: Computed tomography
CT dose index: Computed tomography dose index
TFI: True fidelity image
FBP: Filtered back projection
DLR: Deep learning-based reconstruction
IR: Iterative reconstruction
MBIR: Model-based iterative reconstruction
SNR: Signal-to-noise ratio
CNR: Contrast-to-noise ratio
ALARA: As low as reasonably achievable
