# Supplementary figures and images for: The evaluation of the reduction of radiation dose via deep learning-based reconstruction for cadaveric human lung CT images
Source: Sci Rep. 2022 Jul 20;12:12422. doi: 10.1038/s41598-022-16798-9 (PMC9298173; doi:10.1038/s41598-022-16798-9)

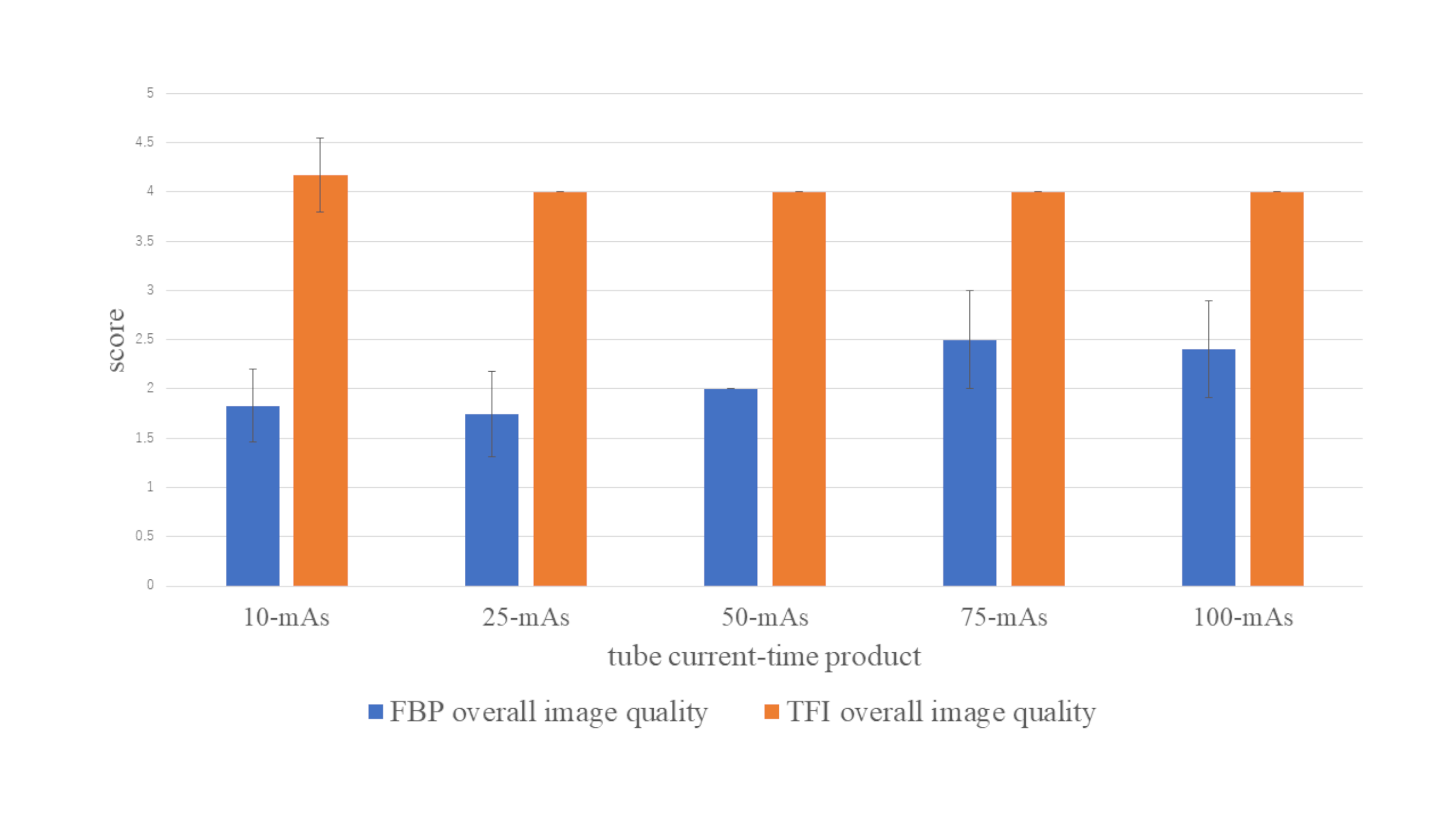

Supplement: Supplementary file 1 — Supplementary Information 1. [file 41598_2022_16798_MOESM1_ESM.tif]

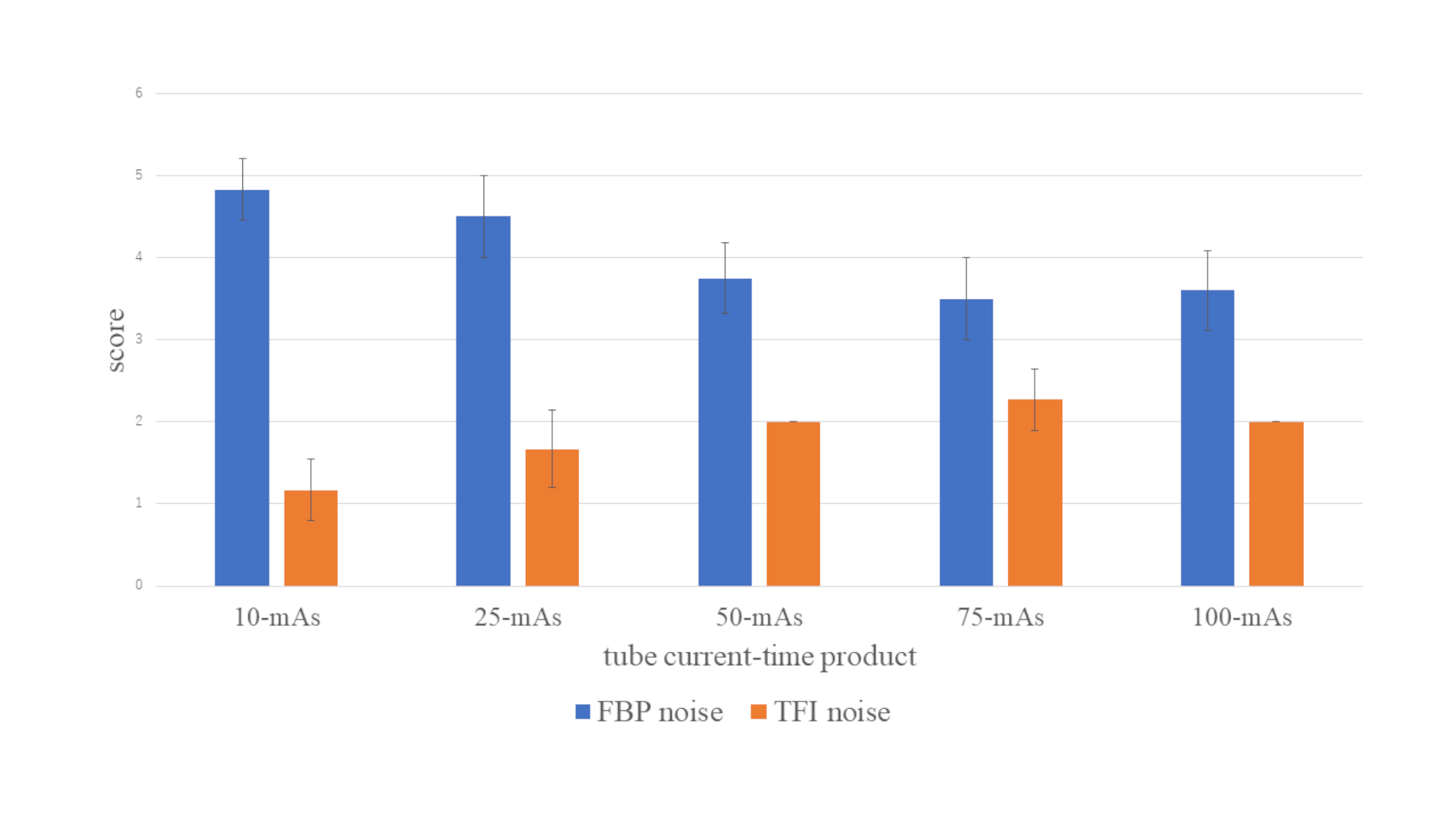

Supplement: Supplementary file 2 — Supplementary Information 2. [file 41598_2022_16798_MOESM2_ESM.tif]

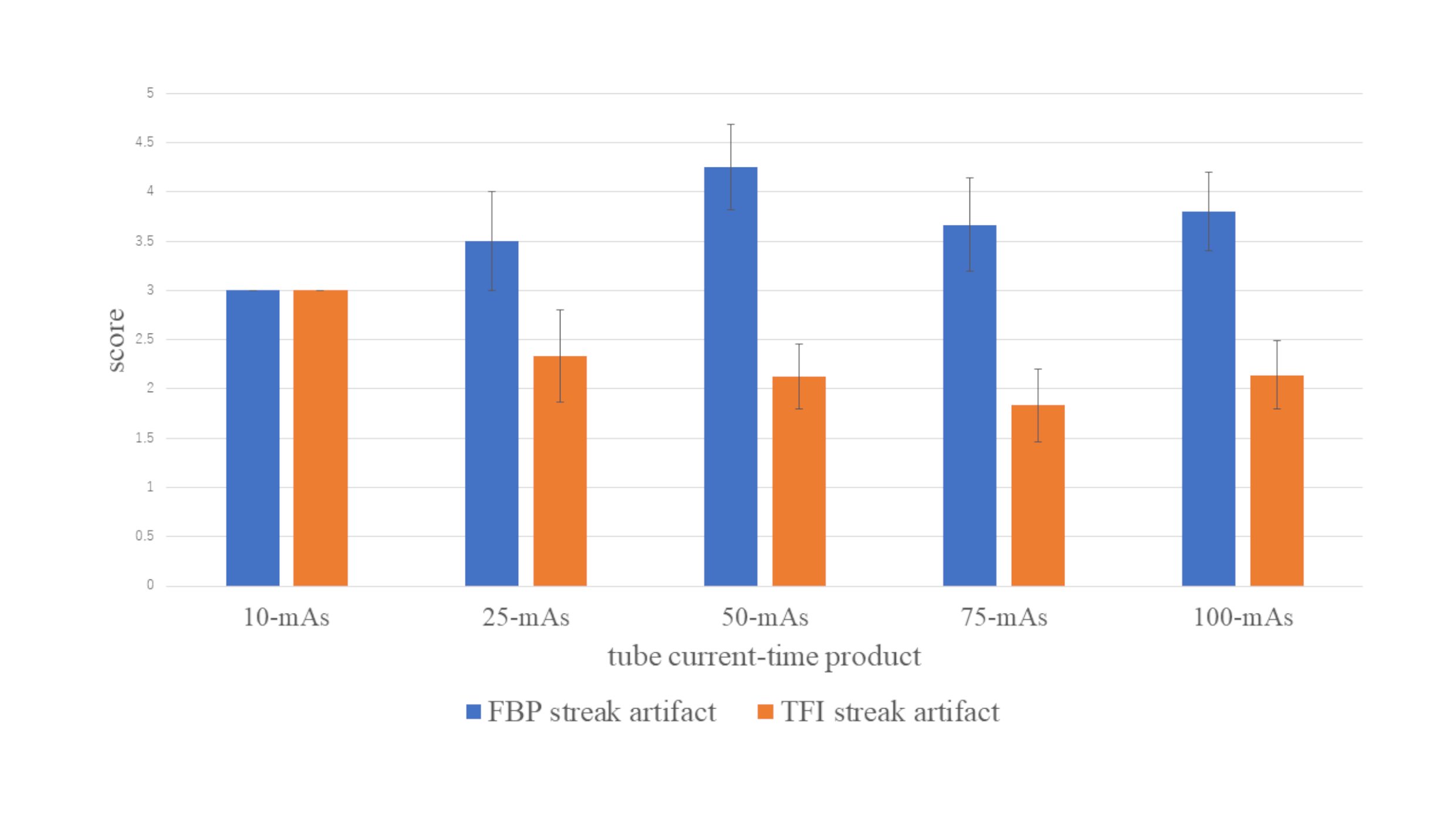

Supplement: Supplementary file 3 — Supplementary Information 3. [file 41598_2022_16798_MOESM3_ESM.tif]

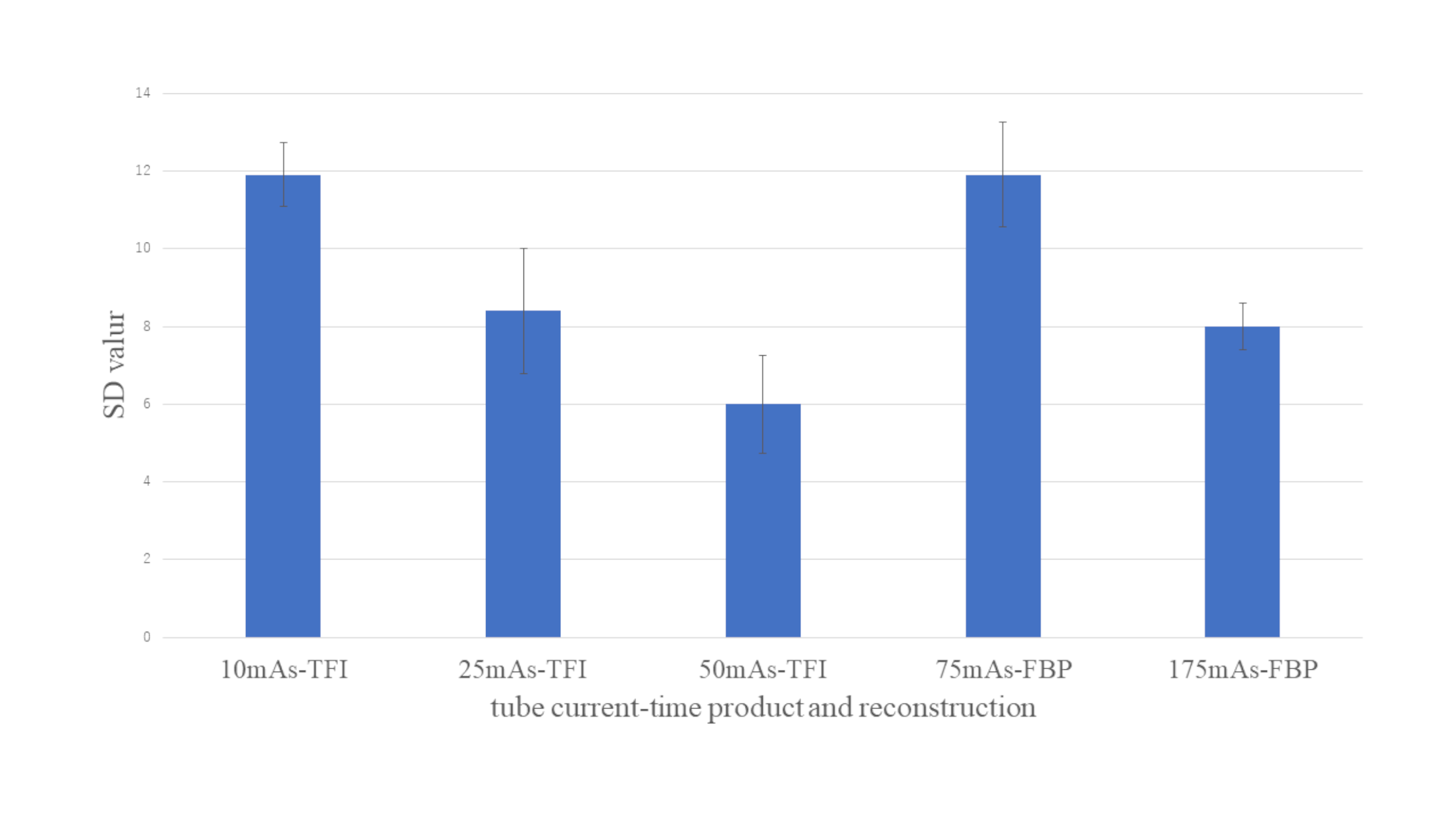

Supplement: Supplementary file 4 — Supplementary Information 4. [file 41598_2022_16798_MOESM4_ESM.tif]
